# A multinational study of the epidemiology, treatment and outcome of childhood arthritis (epoca study): preliminary data from 6,940 patients

**DOI:** 10.1186/1546-0096-12-S1-O8

**Published:** 2014-09-17

**Authors:** Alessandro Consolaro, Pavla Dolezalova, Violeta Panaviene, Anne Estmann Christensen, Rosa Merino, Tamas Constantin, Alberto Martini, Angelo Ravelli

**Affiliations:** 1 Pediatria II, Istituto Giannina Gaslini, Genova, Italy; 2 Paediatrics and Adolescent Medicine, General University Hospital, Prague, Czech Republic; 3 Centre of Pediatrics, Vilnius University, Vilnius, Lithuania; 4 Peadiatric Department, Odense University Hospital, Odense, Denmark; 5 Unidad De Reumatologia Pediatrica, Hospital Universitario La Paz, Madrid, Spain; 6 Unit of Paediatric Rheumatology, Semmelweis University, Budapest, Hungary; 7 Pediatrics, Università di Genova, Genova, Italy

## Introduction

The epidemiology of juvenile idiopathic arthritis (JIA) is known to be variable worldwide and the therapeutic approach to JIA is not standardized. Moreover, the availability of the novel and costly biologic medications is not uniform throughout the world, with possible significant impact on disease prognosis.

## Objectives

The EPOCA study is aimed to obtain information on the frequency of JIA subtypes in different geographic areas, the therapeutic approaches adopted, and the disease status of children with JIA currently followed worldwide.

## Methods

Participation in the study was proposed to the pediatric rheumatology center of all countries belonging to the Pediatric Rheumatology International Trials Organization (PRINTO), and to several centers in the US and Canada. Each centre was asked to enroll 100 consecutive JIA patients or, if less than 100, all consecutive patients seen within 6 months. Each patient received a retrospective and cross-sectional assessment. Parent- and child-reported outcomes were recorded through the administration of the Juvenile Arthritis Multidimensional Assessment Report (JAMAR). Participating countries were grouped into 6 geographic areas.

## Results

Currently, 6,940 patients from 41 countries have been entered in the web database. Comparison of data from the different geographic areas is presented in Table 1.

**Table 1:** 

	Western Europe N = 2845	Eastern Europe N = 2171	Latin America N = 795	Asia N = 726	North America N = 243	Africa N = 79
**Median onset age, yrs**	4	6.3	6.6	5.9	7.5	5.7

**Systemic arthritis, N (%)**	202 (7.1)	165 (7.6)	143 (18)	174 (24)	16 (4.9)	11 (13.9)

**Oligoarthritis, N (%)**	1398 (49.1)	958 (44.1)	247 (31.1)	256 (35.3)	103 (31.8)	25 (31.6)

**Enthesitis related arthritis, N (%)**	253 (8.9)	248 (11.4)	74 (9.3)	92 (12.7)	35 (10.8)	3 (3.8)

**Uveitis, N (%)**	495 (17.4)	232 (10.7)	51 (6.4)	40 (5.5)	38 (11.7)	4 (5.1)

**Median disease activity (JADAS10)**	2	5	3.5	3.5	2	5

**Inactive disease, N (%)**	1070 (37.6)	454 (20.9)	268 (33.7)	237 (32.6)	114 (35.2)	13 (16.5)

**Articular damage (JADI) > 0, N (%)**	352 (12.4)	531 (24.5)	257 (32.3)	136 (18.7)	60 (18.5)	27 (34.2)

**Treated with biologics, N (%)**	1067 (37.5)	637 (29.3)	273 (34.3)	134 (18.5)	178 (54.9)	27 (34.2)

## Conclusion

Patients seen in Western Europe have a younger age at onset and a greater prevalence of uveitis. Systemic arthritis is more common in Asian patients, whereas enthesitis related arthritis is less frequent in African patients. Children from Africa and Eastern Europe have a higher level of disease activity and a lower frequency of inactive disease, and African and Latin American patients have a greater prevalence of articular damage. Biologic medication are administered more frequently in North America and less commonly in Asia.

## Disclosure of interest

None declared.

